# IthaGenes: An Interactive Database for Haemoglobin Variations and Epidemiology

**DOI:** 10.1371/journal.pone.0103020

**Published:** 2014-07-24

**Authors:** Petros Kountouris, Carsten W. Lederer, Pavlos Fanis, Xenia Feleki, John Old, Marina Kleanthous

**Affiliations:** 1 Molecular Genetics Thalassaemia, The Cyprus Institute of Neurology and Genetics, Nicosia, Cyprus; 2 Oxford Radcliffe Hospitals NHS Trust, Oxford, United Kingdom; UMR-S665, INSERM, Université Paris Diderot, INTS, France

## Abstract

Inherited haemoglobinopathies are the most common monogenic diseases, with millions of carriers and patients worldwide. At present, we know several hundred disease-causing mutations on the globin gene clusters, in addition to numerous clinically important *trans*-acting disease modifiers encoded elsewhere and a multitude of polymorphisms with relevance for advanced diagnostic approaches. Moreover, new disease-linked variations are discovered every year that are not included in traditional and often functionally limited locus-specific databases. This paper presents IthaGenes, a new interactive database of haemoglobin variations, which stores information about genes and variations affecting haemoglobin disorders. In addition, IthaGenes organises phenotype, relevant publications and external links, while embedding the NCBI Sequence Viewer for graphical representation of each variation. Finally, IthaGenes is integrated with the companion tool IthaMaps for the display of corresponding epidemiological data on distribution maps. IthaGenes is incorporated in the ITHANET community portal and is free and publicly available at http://www.ithanet.eu/db/ithagenes.

## Introduction

Inherited haemoglobin (Hb) disorders are the most common monogenic diseases, posing a major public health problem worldwide. It is estimated that around 5.2% of the world’s population carry a potentially pathogenic Hb gene and that, annually, over 330 thousand new-borns bear a serious Hb disease [1]. Hb disorders comprise the thalassaemias, sickle-cell disease, Hb E and other, rarer disorders and are prevalent in former malaria regions in the Mediterranean, the Middle East, South-East Asia and Sub-Saharan Africa [2]. However, demographic events, such as migration and the consequent intermixing of populations, have contributed to the spread of Hb disorders worldwide [3], [4]. Therefore, the prevalence of Hb disorders is rising in non-endemic regions, such as Northern and Western Europe and North America, posing a major challenge for researchers and health professionals.

Hb is responsible for binding and transport of oxygen and carbon dioxide by red blood cells and is critical for their shape, integrity and half-life. The Hb protein complex consists of two *α*–like chains, encoded by genes in the *α*-locus (Chromosome: 16, RefSeq ID: NG_000006), namely *ζ* (*HBZ*), *α1* (*HBA1*) and *α2* (*HBA2*), and two *β*-like chains, encoded by genes in the *β*-locus (Chromosome: 11, RefSeq ID: NG_000007), namely *ε* (*HBE*), *Aγ* (*HBG1*), *Gγ* (*HBG2*), *δ* (*HBD*) and *β* (*HBB*). Hb disorders are mainly caused by mutations in the two globin-gene clusters, which can cause defects in the structure of Hb or reduced synthesis of globin chains and of Hb within the red blood cells. In addition, it has been shown that variations in other locations of the genome, such as in the *BCL11A* and *KLF1* genes and the *HBS1L-MYB* intergenic region, have a *trans*-acting effect on globin gene expression and can significantly influence the severity of Hb disorders [5], [6]. Furthermore, numerous phenotypically neutral polymorphisms are utilised as markers for advanced diagnostic approaches, such as non-invasive prenatal diagnosis [7], [8].

Recent advances in biotechnology, particularly the emergence of next-generation sequencing (NGS), have led to an explosion in the amount of genetic information. More specifically, NGS has become a critical tool for identifying medically important variations, which are usually organised and retrieved using variation-centric and genotype-phenotype databases [9], such as dbSNP [10], OMIM [11], ClinVar [12] and HGMD [13]. However, the level of detailed annotation in such databases varies [14], particularly for rare, yet clinically significant, variations.

Besides genome-wide databases, there are numerous locus-specific databases (LSDBs) [15], such as those provided through the Leiden Open Variation Database (LOVD) [16], which lacks a centralised retrieval interface or searching ability across the multiple loci that may effect and affect a specific disease [14]. In the same vein and while LOVD-associated databases are typically curated by experts, there is no centralised policy for quality and content, so that the utility of individual databases is highly variable. Both factors combined make data mining, retrieval and long-term management of detailed disease-specific information across many LSDBs a daunting challenge. Accordingly, curated disease-specific databases have proven a welcome development over the past decade [17] and have been embraced by their respective research fields. Such is the case, for instance, for the AlzGene database for Alzheimer’s disease [18], AutDB for autism [19] and the T1Dbase for type-1 diabetes [20].

In the field of haemoglobinopathies, HbVar [21] is the database with the longest tradition. HbVar provides information about mutations in the globin gene clusters that cause thalassaemia, structural Hb variants or hereditary persistence of foetal haemoglobin (HPFH), while excluding variations in non-globin genes. Although such variations are available through the LOVD Globin Gene Server [22], the globin-related information offered by the latter is only a subset of that stored on HbVar. Moreover and despite recent improvements [23]–[25], the interface functionality, scope for the integration of additional data and user-friendliness of either platform are limited. In particular, incorporation of laboratory, clinical and epidemiological with molecular data is needed to create an integrated resource that could bridge the gap between genetic analysis and clinical practice. Therefore, the absence of a unified, searchable and intuitive database of variations linked to Hb disorders is a persistent problem, not only for researchers but, more importantly, for clinicians and other health professionals.

Herein, we present IthaGenes, a new database that addresses the above shortcomings and provides to the community a universal knowledgebase on Hb disorders. IthaGenes is integrated in the ITHANET community portal [26], an expanding resource for clinicians and researchers dealing with Hb disorders. IthaGenes stores and organises information about genes and variations anywhere in the genome that have been linked to Hb disorders, including disease-causing mutations, disease-modifying mutations and relevant neutral polymorphisms. It provides an easily searchable, user-friendly interface and, where appropriate, renders data graphically to aid comprehension and interpretation. Additionally, IthaMaps, an interactive map tool, was implemented in order to present epidemiological data, which are also embedded in IthaGenes and linked to each sequence variation, when available. Moreover, IthaGenes provides clinical data, such as high-performance liquid chromatography (HPLC) sample images for globin variants, and embeds the National Center for Biotechnology Information (NCBI) Sequence Viewer to display each variation in the context of its latest sequence annotations. IthaGenes is free and publicly available at http://www.ithanet.eu/db/ithagenes, with entries also hyperlinked to IthaMaps, which is available at http://www.ithanet.eu/db/ithamaps.

## Methods

### Data Structure and Management

IthaGenes is freely accessible online for viewing, searching and administrating as a website in the form of HTML documents. The application is written in PHP (http://www.php.net) based on the “Joomla!” content management system (http://www.joomla.org) and uses the jQuery JavaScript library (http://www.jquery.com), as well as packages jQuery-UI (http://www.jqueryui.com), DataTables (http://www.datatables.net) and HighCharts (http://www.highcharts.com), to enhance the presentation of the data. Moreover, IthaMaps utilises the jVectorMap package (http://jvectormap.com) for the visualisation of epidemiological data. The interface does not require the installation of additional plugins, such as Flash and Microsoft Silverlight, and, thus, works across all modern web browsers and the majority of mobile web browsers. All data available in IthaGenes are stored and organised in a relational database using MySQL (http://www.mysql.com), an open-source relational database management system widely utilised in database design in bioinformatics and biomedical informatics. IthaGenes is hosted by the Cyprus Institute of Neurology and Genetics (http://www.cing.ac.cy) using Apache 2 HTTP Server (http://www.apache.org) and is integrated in the ITHANET Community Portal (http://www.ithanet.eu) [26].

### Data Collection and Database Curation

A key component for the creation of a public knowledgebase is the efficient collection, validation and annotation of relevant information. Many of the globin gene causative mutations stored in IthaGenes were initially collated in the books “A Syllabus of Human Hemoglobin Variants (Second Edition)” [27] and “A Syllabus of Thalassemia Mutations” [28], which were the primary sources of information in the initial release of HbVar [21]. In IthaGenes, these mutations were annotated further, while inserting and annotating additional recently reported variations, using more recent articles and reports [6], [29]–[31].

The authors of this study form the IthaGenes Curation Team, responsible for the manual curation of IthaGenes and IthaMaps by adding new and updating existing variations and epidemiological data. Weekly updates on scientific literature are automatically received from PubMed using the following search query: “*thalassemia [tiab] OR thalassaemia [tiab] OR hemoglobin [tiab] OR haemoglobin [tiab] OR sickle-cell [tiab] OR hemoglobinopathies [tiab] OR haemoglobinopathies [tiab]*”. Subsequently, the references are manually filtered to find newly reported variations, recent population studies and recent updates on existing variations. The IthaGenes Curation Team makes every effort to extract information from articles in PubMed that are written in a non-English languages by seeking consultation from members of the advisory committee of the ITHANET community portal or by directly contacting the authors of the corresponding publication. This is also the case for ambiguous cases, such as mutations where the categorisation, location or phenotype is unclear in the publication. Moreover, the curation strategy involves the incorporation of new and updated information from existing public databases, such as HbVar [23], dbSNP [10], ClinVar [12], OMIM [11] and SwissVar [32]. Current lists of members in both the IthaGenes Curation Team and the ITHANET advisory committee can be found in the frequently asked questions. For reasons of data reliability and long-term database maintenance, IthaGenes does not accept direct submissions via its web interface. However, proactive reports of any unpublished or missing variations or frequencies, as well as the suggestion of corrections or improvements are most welcome and can be made via the ITHANET contact form (http://www.ithanet.eu/contact). Contribution of unpublished data will be credited to the contacting scientist on the detailed variation or frequency information page.

## Results

### User Interface and Database Content

**Figure 1:**
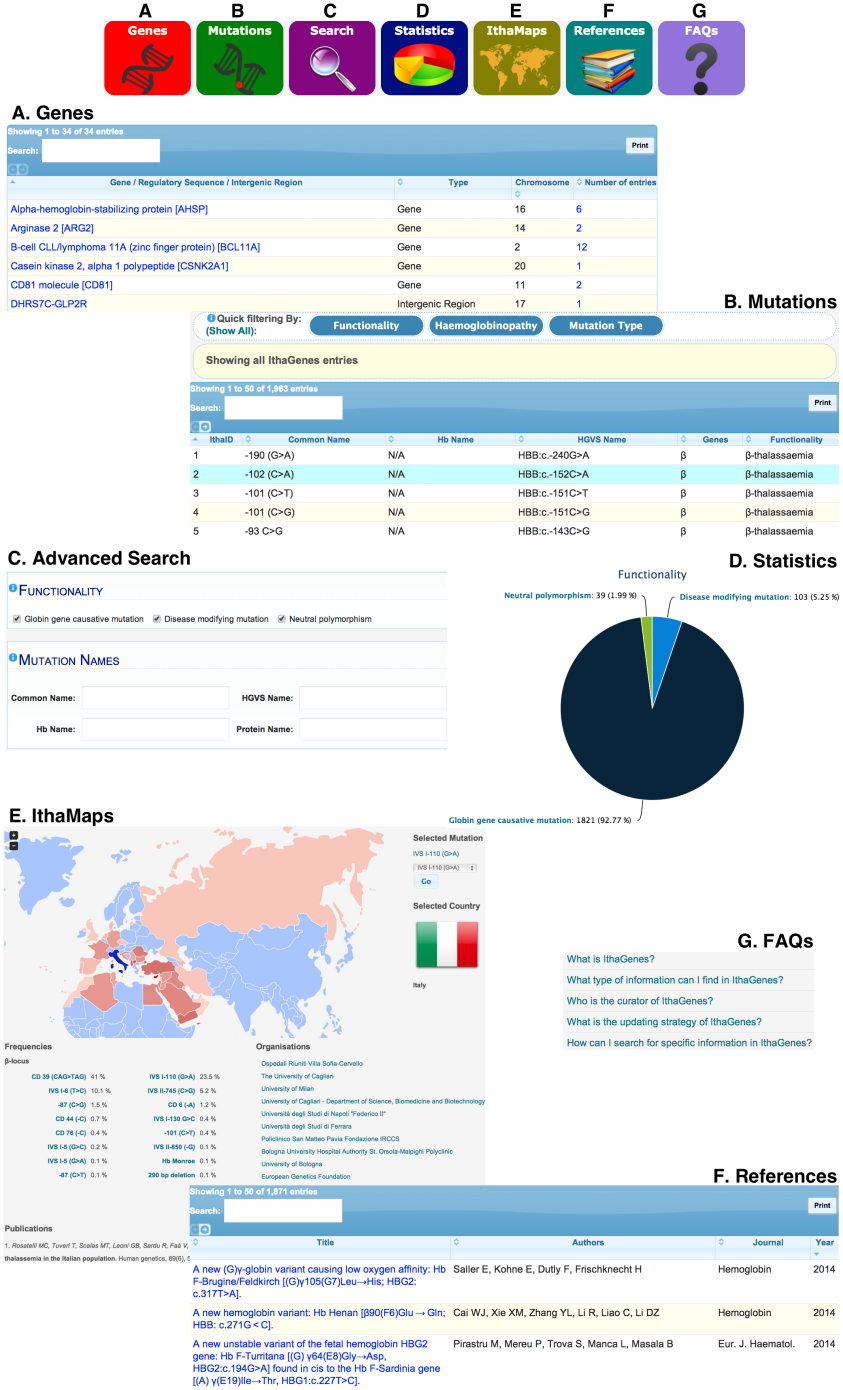
The main functionalities of the IthaGenes database. The six images at the top are displayed on the IthaGenes homepage and link to the seven pages shown below (denoted as sections A, B, C, D, E, F and G). A detailed description for each section is given in the main text.

The IthaGenes homepage displays a general description of the database and the most recent updates and, more importantly, includes links to the main functions of the database, shown in Figure 1: (A) list of genes (*Genes*), (B) list of mutations (*Mutations*), (C) advanced search (*Search*), (D) statistics (*Statistics*), (E) *IthaMaps*, (F) list of references (*References*) and (G) frequently asked questions (*FAQs*). The user can also navigate to the above pages from any other page in the database using the navigation menu that is displayed at the top of each page.

The *Genes* section shows all genes, regulatory sequences and intergenic regions that have at least one IthaGenes entry allocated to them. On 12 June 2014, IthaGenes stored mutations located in 32 different genes, regulatory sequences or intergenic regions, with their distribution shown in Table 1. Mutations on non-globin genes are rarer and have a *trans*-acting effect on the expression of globin genes, such as the levels of foetal haemoglobin (Hb F) [33]. The enormous and still expanding accumulation of data from genome-wide association studies aiming to identify novel modifiers of Hb disorders is set to implicate an increasing number of genes and loci in the determination of Hb disease phenotypes. Importantly, information on *trans*-acting modifiers is already considered as a factor in the clinical management of patients with Hb disorders [34], and the current integration of corresponding data, as implemented in IthaGenes, is therefore critical for the future relevance of Hb-related databases as a clinical resource.

**Table 1:** The list of genes, regulatory sequences and intergenic regions available in IthaGenes on 12 June 2014.

Name	Type	Chr	No. of entries
HS40	Regulatory Sequence	16	41
HBZ (ζ)	Gene	16	49
HBA2 (α2)	Gene	16	280
HBA1 (α1)	Gene	16	192
HBA1 or HBA2 (α1 or α2)	Gene	16	188
α3.7 hybrid	Gene	16	10
LCRB	Regulatory Sequence	11	29
HBE (ε)	Gene	11	23
HBG2 (Gγ)	Gene	11	96
HBG1(Aγ)	Gene	11	88
HBG1 or HBG2 (Gγ or Aγ)	Gene	11	3
HBBP1 (pseudo β)	Pseudogene	11	45
HBD (δ)	Gene	11	172
HBB (β)	Gene	11	991
BCL11A	Gene	2	12
HBS1L – MYB	Intergenic Region	6	18
KLF1	Gene	19	31
FRMPD4	Gene	X	1
PHEX	Gene	X	2
LOC389842– MAGEB18	Intergenic Region	X	1
CSNK2A1	Gene	20	1
HMOX1	Gene	22	1
CD81	Gene	11	2
DHR57C – GLP2R	Intergenic Region	17	1
EIF2S3	Gene	X	1
HAO2	Gene	1	1
PDE7B	Gene	6	5
TOX	Gene	8	7
NOS1	Gene	12	2
FLT1	Gene	13	6
ARG2	Gene	14	2
NOS2A	Gene	17	2
MAP3K5	Gene	6	2
AHSP	Gene	16	6

The most recent list can be found at the IthaGenes website (http://www.ithanet.eu/db/ithagenes?action=glist).

**Figure 2:**
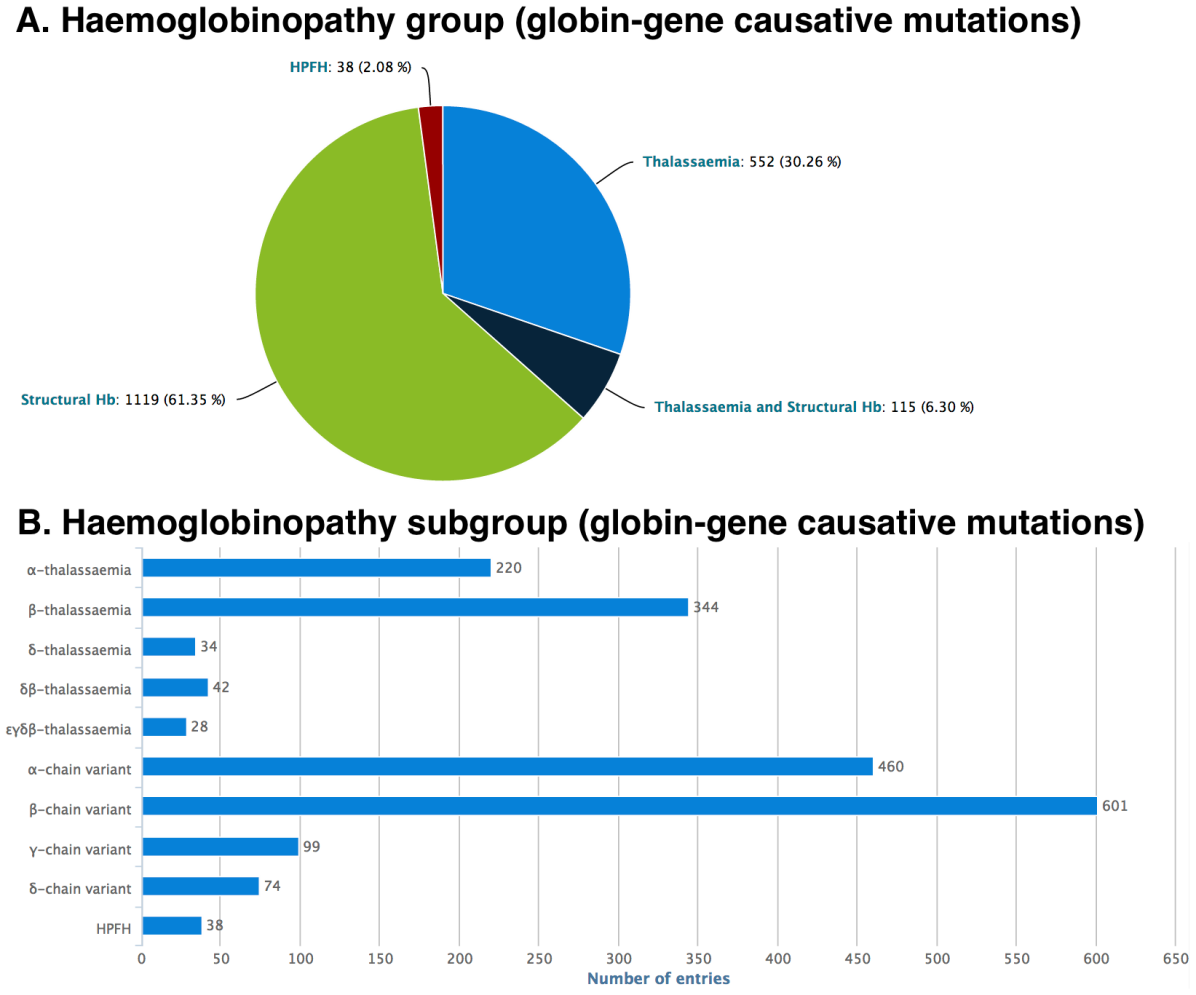
Distribution of globin-gene causative mutations stored in IthaGenes on 12 June 2014, based on the assigned haemoglobinopathy group (panel A) and the haemoglobinopathy subgroup (panel B). Current distributions and other statistics can be found at http://www.ithanet.eu/db/ithagenes?action=stats.

The *Mutations* section shows all mutations and variations stored in IthaGenes. On 12 June 2014, IthaGenes stored 1963 mutations or variations reported in 1751 individual publications, including original publications, review articles and textbooks. Each IthaGenes entry is assigned to a single functional category: (i) globin gene causative mutation, (ii) disease-modifying mutation or (iii) neutral polymorphism and, for each functional category, a custom list of parameters is collected, shown in Table 2. In addition, each globin gene causative mutation is assigned to a single haemoglobinopathy group: (i) Thalassaemia, (ii) Structural Hb (including disease-causing variants, such as HbS and HbE) (iii) Thalassaemia and Structural Hb or (iv) HPFH and, subsequently, it is assigned to one or more haemoglobinopathy subgroups. Distribution of IthaGenes entries based on haemoglobinopathy group and subgroup are shown in Figure 2. Detailed information about the current IthaGenes content is displayed graphically and in real time in the statistics section of the web site (http://www.ithanet.eu/db/ithagenes?action=stats; see Figure 1, Section D for an example).

**Table 2:** The list of parameters collected for each IthaGenes entry based on its functional category.

	Functional Categories		
Parameter Name	A	B	C
Functionality	•	•	•
Common name	•	•	•
HGVS name	•	•	•
Haemoglobin name	•		
Protein name	•	•	
Ref Allele	•	•	•
Strand	•	•	•
Context sequence	•	•	•
Protein sequence	•	•	•
Synonyms	•	•	•
Comments	•	•	•
Chromosome	•	•	•
RefSeq locus	•	•	•
RefSeq locus location	•	•	•
Size	•	•	•
Located at <select gene/regulatory sequence/intergenic region>	•	•	•
Specific location <select intron/exon etc.>	•	•	•
Haemoglobinopathy group	•		
Haemoglobinopathy subgroup	•		
Allele phenotype for haemoglobinopathies	•		
Allele phenotype for non-globin genes (*cis*- acting and *trans*-acting)		•	•
Clinical phenotype	•	•	•
Ethnic origin	•	•	•
Type of mutation	•	•	•
Effect on gene/protein function	•	•	
Inheritance	•	•	•
DNA sequence/breakpoint determination	•	•	•
Detection methods	•	•	•
Publications	•	•	•
Links	•	•	•

(A: Globin-gene causative mutation; B: Disease-modifying mutation; C: Neutral polymorphism).

The *References* section (Figure 1, Section F) shows all publications used to add and annotate genes and variations in IthaGenes and IthaMaps, hyperlinked to the corresponding abstract in PubMed. Thus, a user can locate whether a publication is missing from the database and use the *Contact us* subject “New publication” to have it added to the database by the IthaGenes Curation Team. Up to 12 June 2014, 1871 publications have been used for annotation in IthaGenes and IthaMaps.

A critical component for the success of a biological database is a clear and comprehensive help section. IthaGenes provides an expanding help section in the form of frequently asked questions (Figure 1, Section G), which describe the main parts of the database and clarify possible misconceptions. IthaGenes users can ask new questions or suggest improvements to the database through the ITHANET contact form (http://www.ithanet.eu/contact). In addition, short descriptions for specific parts or fields in the database are provided in the form of pop-up tooltips, which are shown by placing the mouse over the information icon (denoted with an “i” icon) that appears throughout the database interface.

### Searching IthaGenes

IthaGenes facilitates finding required information by offering three different search options: (i) live search, (ii) quick filtering and (iii) advanced search. First, each table (i.e. the list of genes, the list of mutations and the list of references; Figure 1, Sections A, B and F) offers live searching, i.e. dynamic filtering of the table content while typing. This option is automatically applied to all visible fields of the *Genes, Mutations* or *References* tables and is particularly useful when a user is looking for a specific mutation or gene based on its name or for a reference by its title or by an author’s surname. Second and to also filter for additional database content, the *Mutations* list can be filtered using the “Quick Filtering” menu located above the table (see Figure 1, section B), which includes pre-defined searches of the most common search queries, based on the functionality, haemoglobinopathy and mutation type of each entry. Finally, most of the data available in IthaGenes are searchable through the advanced search section (see Figure 1, Section C), which can be utilised to search for every possible field combination. The advanced search option offers great flexibility and facilitates displaying and printing of custom datasets based on a variety of user-defined field combinations.

### IthaMaps

IthaMaps (http://www.ithanet.eu/db/ithamaps) is an interactive map tool that displays epidemiological data concerning Hb disorders. It is accessible as a stand-alone application for the visualisation of epidemiological data worldwide, while it is also embedded in IthaGenes for specific mutations. On 12 June 2014, IthaMaps included epidemiological data for 127 globin gene causative mutations in 56 countries, extracted from 89 individual publications. Although some of the reported data are extracted from old publications, the information stored in IthaMaps are considered the best available epidemiological information in many countries in a recent textbook by the Thalassaemia International Federation [30].

IthaMaps has two main display modes: (i) worldwide distribution of relative frequencies for a selected mutation and (ii) epidemiological data for a selected country. When a user selects a mutation from the drop-down menu to the right of the interactive map, the relative frequencies for each country are visually displayed on the map. The exact value for each country is shown on the pop-up tooltip that appears when the user puts the mouse over the country of interest. By clicking on a country, information about the specific country is displayed, including relative frequencies for different mutations in the country, with a link to the detailed description of each mutation in IthaGenes, as well as relevant publications. Additionally and where available, a list of organisations related to Hb disorders in the country is shown, with a link to the detailed description of the organisation in the ITHANET portal. The example shown in Figure 1 (Section E) demonstrates the functionality of IthaMaps by showing the worldwide distribution of a selected mutation (e.g. IVS I-110 G>A) as well as information (frequencies, organisations and publications) for a selected country (e.g. Italy), shown in blue on the freely resizable map.

### Viewing IthaGenes Entries

IthaGenes provides a detailed description for variations, genes, regulatory sequences and intergenic regions, which includes detailed information about each entry, external links, references, the embedded NCBI Sequence Viewer and a complete revision history.

First, detailed information about a specific IthaGenes variation is shown after clicking on the corresponding row of the *Mutations* table. The information shown for each entry varies based on its functionality and the data availability. The detailed view of an IthaGenes entry comprises nine sections: (i) *Names and Sequences*, (ii) *Links*, (iii) *Location*, (iv) *Phenotype*, (v) *Other details*, (vi) *HPLC*, (vii) *Sequence Viewer*, (viii) *Frequencies* and (ix) *Publications/Origin*. Sections (vi) and (viii) are shown only when relevant data are available, i.e. HPLC images and IthaMaps epidemiology data, respectively. On 12 June 2014, HPLC images, provided by Bio-Rad Laboratories Inc., were available for 73 structural Hb variants, while IthaGenes provided 4869 links to five different external databases, namely dbSNP [10] (1740 links), HbVar [23] (1608 links), OMIM [11] (880 links), SwissVar [32] (487 links) and ClinVar [12] (155 links).

An important component of the IthaGenes interface is the embedded NCBI Sequence Viewer, which offers a visual representation of each IthaGenes entry at its corresponding location and its interconnection with other NCBI resources [35]. The NCBI Sequence Viewer was selected over other genome browsers because of its simplicity and the ability to embed it in any page without the need for local installation and administration [36]. The simplicity of the interface is crucial for clinicians and health professionals who are not familiar with complex applications currently available in biological research. In addition to the standard tracks shown in the NCBI Sequence Viewer, a custom track (entitled “IthaGenes”) shows all sequence variations stored in IthaGenes with links to their IthaGenes detailed page and other external databases.

**Figure 3:**
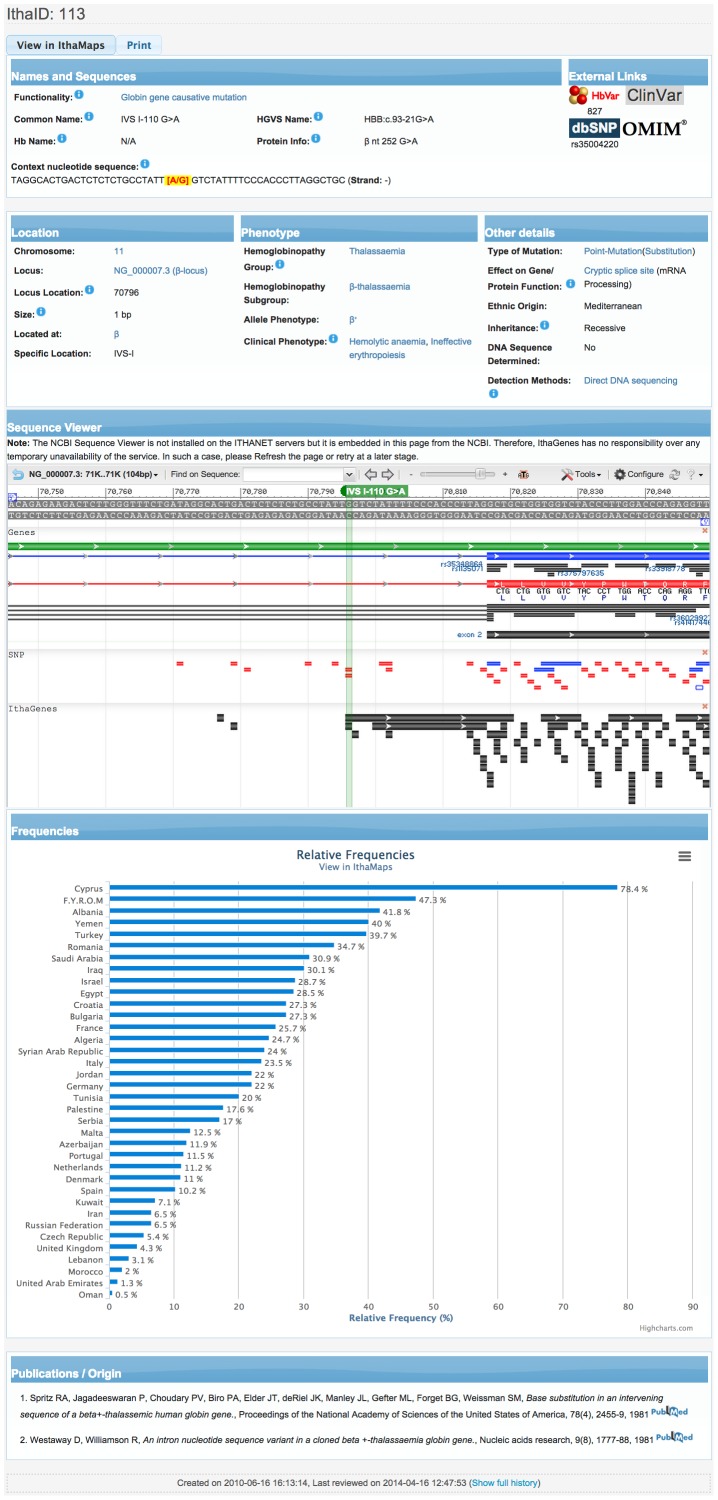
The detailed view of an IthaGenes entry, IVS I-110 (G>A) (http://www.ithanet.eu/db/ithagenes?ithaID=113). Details for each section are given in the main text.

Figure 3 shows the detailed description of one of the most common forms of β-thalassaemia in the Mediterranean population, IVS I-110 G>A, which is one of the best-studied mutations in terms of epidemiology. By clicking on the country name, the user can navigate to IthaMaps and view the epidemiological information. Moreover, other IthaGenes entries display information about HPLC data, as is the case for the β-chain variant Hb Hikari (http://www.ithanet.eu/db/ithagenes?ithaID=1005), which demonstrates the way HPLC images are displayed in PNG format, while the detailed report can be downloaded in PDF format.

**Figure 4:**
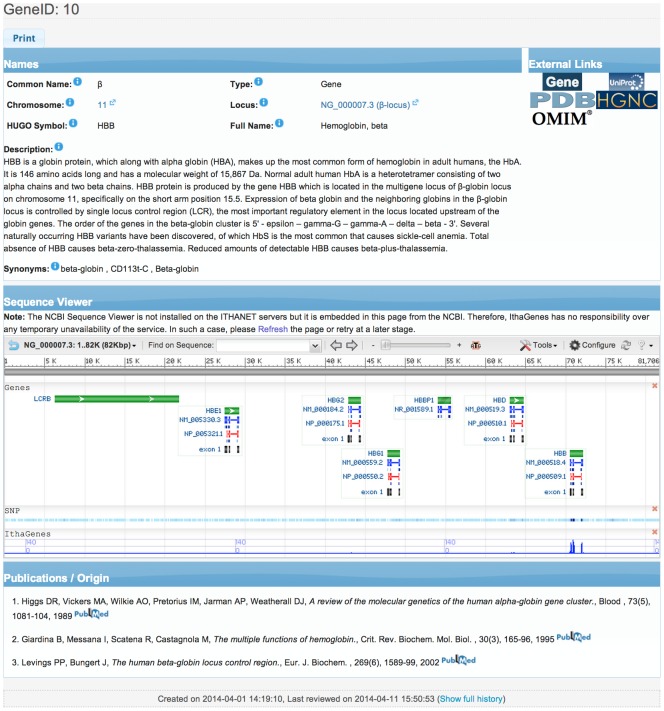
The detailed description for globin gene (HBB) in IthaGenes (http://www.ithanet.eu/db/ithagenes?geneID=10).

In addition, detailed information about a specific gene, regulatory sequence or intergenic region is shown after clicking on the corresponding name of the *Genes* table. More specifically, information about the official Human Genome Organisation (HUGO) name and symbol and other synonyms is provided as well as the chromosome and locus of the sequence, with links to the corresponding nucleotide sequence in NCBI GenBank [37], while a detailed description of the functionality of the gene and its role in inherited haemoglobinopathies is shown. Moreover, links to external databases are provided, such as NCBI Gene [35], UniProtKB [38], OMIM [11], HGNC [39] and PDB [40], as well as related publications hyperlinked to NCBI PubMed, while the corresponding locus is shown on the embedded NCBI Sequence Viewer [35]. Figure 4 shows the detailed description of the β-globin gene in IthaGenes.

## Discussion

IthaGenes is an interactive archive of genes and variations affecting haemoglobin disorders, including the globin loci, disease modifiers and other significant variations. IthaGenes stores and organises phenotype, epidemiology, HPLC data, as well as related publications and external links, while embedding the NCBI Sequence Viewer in the website for detailed graphical representation of each entry. Retrieving information is facilitated through live search and pre-defined filters, while most of the fields stored in the database are also specifically searchable by advanced search.

Specifically comparing with HbVar, the longest-established database in the field, on 12 June 2014, IthaGenes stored the vast majority of the disease-causing mutations stored in HbVar (1593 out of 1604), while providing 228 mutations that were not reported in HbVar. In addition to those globin-gene causative mutations, IthaGenes reported 142 disease-modifying mutations and neutral polymorphisms that were not stored in HbVar. That last category in particular is expected to increase in numbers and in its significance for the genotype-phenotype correlation for haemoglobinopathies over the coming years and, thus, marks a qualitative rather than just quantitative difference of IthaGenes as an integrated database. Therefore, IthaGenes, universally covering the human genome for genes and variations related to Hb disorders, represents a significant advancement over existing databases of Hb variations in its content, while offering improved searching ability, display functionality, true integration with additional resources, such as the NCBI genome browser and IthaMaps, and a user-friendly interface. Hence, we expect IthaGenes to be established not only as a useful tool for researchers in the field of Hb disorders, but also as an important resource for the prevention and diagnosis of inherited haemoglobinopathies.

Future improvements of IthaGenes include the incorporation of more detailed information about the clinical phenotype observed for each variation, sequence-based search functionality and the inclusion of a larger number of trans-acting genes in the database. Moreover, proactive cross-exchange and sharing of annotated data with other databases, such as dbSNP [10], UniProtKB [38] and HbVar [25], is envisioned for the future. In addition, we aim to develop IthaMaps further in order to include more detailed and recent epidemiological information and incorporate data not only at national but also at subnational level. With this, we encourage scientists that have updated or more detailed epidemiological information to contact us in order to improve the quality and content of IthaMaps for the benefit of the haemoglobinopathy community.

IthaGenes is integrated in the ITHANET portal [26], a website dedicated to Hb disorders and an instrument for dissemination of information about Hb disorders to researchers, clinicians and patients. The ITHANET Portal is a growing public resource on Hb disorders and represents the ideal place to host such a database in order to ensure its consistent update and expansion. ITHANET is maintained by permanent funding, thus ensuring long-term development of the database, and is an active partner in and beneficiary of primary research data from several projects on Hb disorders. Facilitated through on-going collaborations and as a milestone for long-term database development, IthaGenes is the first step towards the implementation of a complete genotype-phenotype database on haemoglobinopathies.
